# Fusion between Hematopoietic and Epithelial Cells in Adult Human Intestine

**DOI:** 10.1371/journal.pone.0055572

**Published:** 2013-01-30

**Authors:** Alain D. Silk, Charles E. Gast, Paige S. Davies, Farnaz D. Fakhari, Gretchen E. Vanderbeek, Motomi Mori, Melissa H. Wong

**Affiliations:** 1 Department of Dermatology, Oregon Health & Science University, Portland, Oregon, United States of America; 2 Knight Cancer Institute, Oregon Health & Science University, Portland, Oregon, United States of America; 3 Program in Cell and Molecular Biology, Oregon Health & Science University, Portland, Oregon, United States of America; 4 Department of Public Health & Preventive Medicine, Oregon Health & Science University, Portland, Oregon, United States of America; University Claude Bernard Lyon 1, France

## Abstract

Following transplantation of hematopoietic lineage cells, genetic markers unique to the transplanted cells have been detected in non-hematopoietic recipient cells of human liver, vascular endothelium, intestinal epithelium and brain. The underlying mechanisms by which this occurs are unclear. Evidence from mice suggests it is due in part to fusion between cells of hematopoietic and non-hematopoietic origins; however, direct evidence for this in humans is scant. Here, by quantitative and statistical analysis of X- and Y-chromosome numbers in epithelial and non-epithelial intestinal cells from gender-mismatched hematopoietic cell transplant patients, we provide evidence that transplanted cells of the hematopoietic lineage incorporate into human intestinal epithelium through cell fusion. This is the first definitive identification of cell fusion between hematopoietic cells and any epithelial cell type in humans, and provides the basis for further understanding the physiological and potential pathological consequences of cell fusion in humans.

## Introduction

In patients who received hematopoietic cell transplantation, genetic markers specific to transplanted hematopoietic lineage cells have been found in fully differentiated cells of multiple non-hematopoietic tissues, including liver, brain, vascular endothelia, intestinal epithelia and cancerous tissue [1]–[5]. Despite this overwhelming evidence, it is not currently known how it occurs in the vast majority of cases [4]. Indeed, there is considerable debate as to the mechanisms underlying the presence of hematopoietic-specific genetic markers in non-hematopoietic human tissue. One possibility is that transplanted hematopoietic stem cells (HSCs) retain a high degree of plasticity, and after homing to non-hematopoietic cellular compartments undergo transdifferentiation into cell types outside of the recognized hematopoietic lineage. The alternative is that transplanted HSCs or HSC-derived cells undergo direct cell fusion with non-hematopoietic cell types, producing hybrids carrying genetic material from both parental cells. It is probable that these two mechanisms have distinct consequences for tissue physiology. Hematopoietic transdifferentiation, involving the reprogramming of a single genome, is more likely to produce cells that are phenotypically similar to resident differentiated cells within a given tissue. Cell fusion, by virtue of combining two differentially regulated genomes within a single cell, has a greater potential to produce cells that show fundamentally different behaviors relative to surrounding tissue-resident cells. While there have been repeated demonstrations in humans that genetic markers specific to hematopoietic cells can be found in non-hematopoietic cell types, there have been very few attempts to conduct quantitative analysis at the single-cell level to definitively identify whether this occurs via hematopoietic transdifferentiation or cell fusion. Distinguishing between these mechanisms is necessary in order to guide subsequent investigation towards the plasticity of hematopoietic progenitor cells or the phenotypic outcomes of fusion between different cell types. One obvious feature that distinguishes cells derived from fusion relative to transdifferentiation as a mechanism for the origin of non-hematopoietic cells carrying hematopoietic-specific genetic markers is that cell fusion results in a direct and immediate increase in cellular chromosomes content, while transdifferentiation does not.

In the human brain, there is strong support for cell fusion as one mechanism by which markers from transplanted hematopoietic cells incorporate into non-hematopoietic recipient tissue. There is an abnormally high number of X-chromosomes in Y-chromosome-containing Purkinje neurons in female recipients of gender-mismatched bone marrow transplantation; consistent with hematopoietic-Purkinje fusion in the cerebellum [4]. These observations are supported by studies in mice, which demonstrate that bone marrow-derived cells undergo fusion with cerebellar Purkinje neurons [6], [7]. By contrast, incorporation of hematopoietic-specific genetic markers into endothelium appears to occur exclusively by transdifferentiation, in both humans and mice [1], [8]. However, in other human tissues it is not known how genetic markers unique to hematopoietic cells come to exist in non-hematopoietic cell types. For example, while fusion between hepatocytes and hematopoietic lineage cells has been conclusively demonstrated in mice, there is conflicting evidence as to whether it also occurs in humans [9]–[11]. Similarly, while spontaneous cell fusion between hematopoietic and non-hematopoietic cells in a wide variety of other tissues is supported by multiple studies in mice, there has been considerable debate as to whether it occurs in humans at all [12]–[14].

In the human gastrointestinal tract, it is clear that donor-specific markers are found within the epithelium of hematopoietic cell transplant patients [2], [15]. Evidence from mice suggests that cell fusion plays a part in this process and transcriptional analysis of fusion-derived cells indicates that these cells share transcriptional characteristics of both epithelial and bone marrow-derived myeloid cells [14], [16]. In addition, there is a detectable basal level of hematopoietic-epithelial fusion in the mouse intestine in the absence of irradiation-induced injury, indicating that fusion occurs endogenously and independent of cellular transplantation [17]. These results suggest that hematopoietic-epithelial cell fusion may contribute to aspects of intestinal pathophysiology or potentially the replacement of epithelial cells lost by continuous sloughing of the epithelial cell layer, but previous studies have failed to find evidence of cell fusion in the human intestine [18]. Here, by quantitative and statistical analysis of X- and Y-chromosome numbers in individual epithelial and non-epithelial nuclei of gender-mismatched hematopoietic cell transplant patients, we demonstrate that cell fusion is one mechanism by which hematopoietic lineage cells incorporate into the human gastrointestinal epithelium.

## Materials and Methods

### Human tissues samples and ethics statement

Screening the Oregon Health & Science University (OHSU) hematopoietic transplantation registry identified one-hundred and ninety-five female patients who had received hematopoietic cell transplant from male donors between 1994 and 2011. Of these, thirty-six were diagnosed with acute graft-versus-host disease (GVHD) and underwent skin and intestinal biopsies for confirmation of GVHD diagnosis by medical pathologists. Patients without suspected GVHD are not biopsied. In each biopsy sample pathology reports confirmed the GVHD diagnosis. We acquired intestinal tissue sections from ten of these patients for analysis in this study and classified GVHD as mild, moderate or severe in each patient depending on the degree of crypt necrosis, confluent apoptosis, and/or heavy inflammatory infiltrate. Two patients were excluded due to moderate or severe GVHD with disorganized intestinal histology, making the epithelial cell population difficult to identify. Fluorescence *in situ* hybridization (FISH) and immunohistochemical staining failed on tissues from two additional patients, and therefore these were not included in our analysis. Human tissue samples were collected according to the ethical requirements and regulations of the OHSU institutional review board (IRB; FWA00000161) with written consent provided by patients. Approval to use de-identified archived tumor tissues in this study was provided by the OHSU IRB under approved protocol number IRB5169.

### Fluorescence *in situ* Hybridization

X- and Y-chromosome FISH probes were hybridized to 5 µm paraffin intestinal tissue sections. Briefly, tissue sections were deparaffinized, treated with Retrievagen A (BD Biosciences, CA) and processed with the Tissue Digestion Kit II reagents (Kreatech, Netherlands) according to the manufacturer's protocol. CEP X (DXZ1 locus) and Y (DYZ1 locus) probes (Abbott Molecular, IL) were hybridized to samples at 80°C for 5 min, followed by incubation at 37°C for 12 h. Samples were washed in 2×SCC (30 mM sodium citrate, 300 mM sodium chloride, pH 7.0)+0.1% NP-40 at 24°C for 2 min, 2×SCC+0.3% NP-40 at 72°C for 5 min, and 2×SCC+0.1% NP-40 at 24°C for 1 min. Tissue sections were dehydrated with a series of graded alcohols, air dried, and washed twice in phosphate buffered saline (PBS) for 5 min prior to antibody staining.

### Immunofluorescence Staining and Microscopy

FISH-processed intestinal tissues were incubated in Blocking Buffer (PBS +10% Donkey Serum, 5% bovine serum albumin +0.3% TritonX-100) for 30 min at 24°C followed by incubation with goat-anti-human Lamin B1 antibodies (1∶200, Santa Cruz Biotechnology, CA) in a humidified chamber at 4°C for 12 h. The Lamin B1 antibody was visualized by incubation with a Cy5-conjugated anti-goat antibody (1∶2000; Jackson, PA) at 24°C for 1 h. Coverslips were mounted with ProLong Gold antifade reagent (Invitrogen, NY). Staining for cytokeratin was performed by removing coverslips from FISH and Lamin B1 stained sections, and incubating with guinea pig anti-Cytokeratin 14 antibody (1∶100, Fitzgerald, MA) followed by an Alexa 488- conjugated anti-guinea pig secondary antibody (1∶200, Life Technologies, NY). Tissue sections were imaged using a Zeiss LSM780 confocal microscope mounted on a fully motorized AxioObserver Z1 inverted microscope stand, controlled by ZEN2009 software (Carl Zeiss, NY). 1 µm-thick optical sections were captured in 1.5 µm intervals spanning the entire thickness of each tissue section. Maximum intensity projections of Lamin B1-positive planes were generated for manual counts of X- and Y-chromosome signals.

### Immunohistochemical Staining

For detection of FABP2/IFABP, tissue sections were deparaffinized and subjected to 50 minutes of boiling in 10 mM Sodium Citrate pH 6 with 1 mM EDTA, followed by 5 minutes in 2% H_2_O_2_ in methanol. Slides were incubated for 30 minutes in Blocking Buffer and then overnight with a rabbit-anti-FABP2 antibody (1∶200, Sigma, MO), followed by incubation with a biotinylated goat anti-rabbit secondary antibody (1∶500, Jackson ImmunoResearch, PA). Diaminobenzidine staining was carried out with the Vectastain ABC and Peroxidase Substrate DAB kits (Vector Laboratories, CA). Hematoxylin (Vector Laboratories, CA) was applied as a counterstain.

### Image Scoring, Quantification and Statistics

More than 20,000 Lamin B1-stained epithelial cells from female GVHD patients who had previously received male hematopoietic cell transplant were screened for the presence of a Y-chromosome. The total number of epithelial cells screened was calculated by determining the average number of epithelial cells in eight acquired microscope fields of view (≈300 cells per field), including at least one field from each patient, and multiplying by the total number of fields examined (n = 74). The number of X-chromosomes was counted in each intestinal cell nucleus with a contiguous Lamin B1-stained nuclear envelope and a single Y-chromosome. We classified cells as epithelial or non-epithelial using histological criteria, based on the spatial organization of nuclei within the intestine, as conventionally histologically defined [19]. We also determined the number of X-chromosomes in Y-chromosome positive cells from epithelial and adjacent non-epithelial compartments in sections of normal male intestinal tissue. Quantification of chromosome numbers in cells from GVHD and control tissues was validated by blinded re-count. Fisher's exact test was performed to test for significant differences between groups, regarding the incidence of cells with 2 or 3 X-chromosomes; a p-value of less than 0.05 was considered statistically significant.

## Results

**Figure 1:**
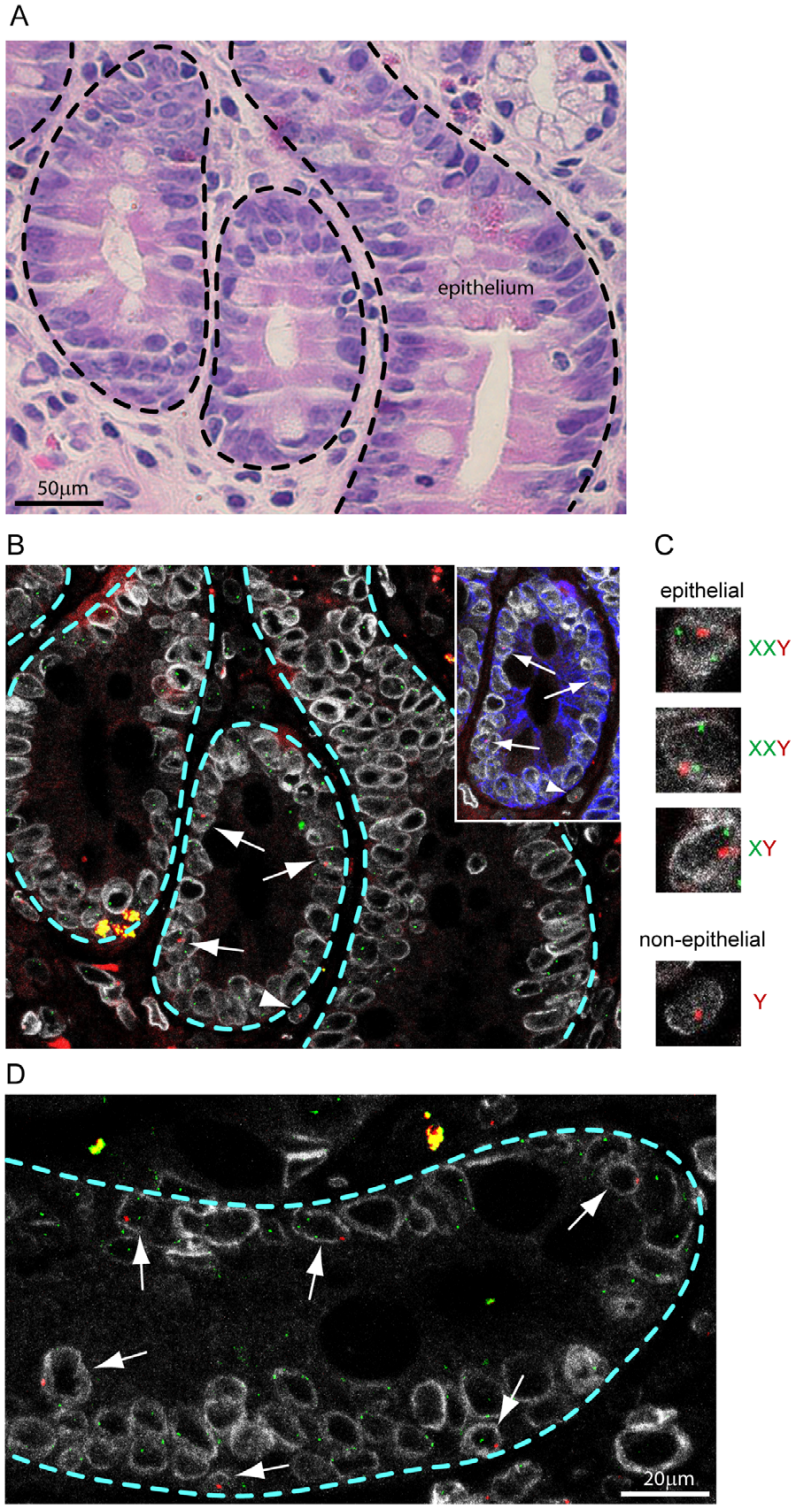
Epithelial compartmentalization and sex-karyotyping of intestinal cells. (A) Hematoxylin and Eosin stained intestinal biopsy; epithelial compartment is labeled. (B) Adjacent tissue section to that from panel A stained for X- (green) and Y- (red) chromosomes and Lamin B1 (white). Arrows indicate Y-chromosome-positive epithelial cells and the arrowhead points to a Y-chromosome-positive non-epithelial cell. Inset shows a sub-region stained for cytokeratin (blue); arrows and arrowhead serve as positional references. (C) Enlarged views of cells indicated in panel B by arrows and arrowhead; sex-karyotype is indicated for each. (D) Independent patient sample also stained for X- (green) and Y- (red) chromosomes and Lamin B1 (white). Arrows indicate Y-chromosome-positive epithelial cells. Dashed lines in all panels indicate boundaries of epithelial and non-epithelial compartments.

In this study we demonstrate that cell fusion is one mechanism by which genetic markers of hematopoietic cells are incorporated into human intestinal epithelium. To do this, we analyzed intestinal biopsies from female patients who had received gender-mismatched peripheral blood stem cell or bone marrow transplantation (Table 1). Fusion between hematopoietic cells and intestinal epithelial cells is known to occur in mice but has not yet been identified in humans [14], [16], [17].We chose to analyze samples from male-into-female gender-mismatched transplant patients because this situation provides a genetic marker—the Y-chromosome—which uniquely identifies putative fused or transdifferentiated hematopoietic cells with single cell resolution. All of our samples were from patients diagnosed with GVHD because only patients with suspected cases of GVHD underwent intestinal biopsy; GVHD was confirmed by medical pathologists in each case. No intestinal biopsy samples were available from gender-mismatched transplant patients that did not also have a confirmed GVHD diagnosis. We sub-classified GVHD in each case as mild, moderate or severe based on the degree of inflammation and disruption of normal intestinal tissue architecture. We conducted analysis only on patient samples with mild or moderate GVHD in which epithelial and non-epithelial intestinal compartments could be readily distinguished. Fluorescent *in situ* hybridization (FISH) for X- and Y-chromosomes was used to identify Y-chromosome-containing nuclei within the epithelial and non-epithelial compartments of the intestine and quantify the number of X-chromosomes in each of these nuclei. Nuclear boundaries were identified by the presence of Lamin B1. The epithelium of the intestine comprises a histologically distinct compartment defined by basal localization and positioning of nuclei within each tissue section [19]. Epithelial classification of cells was guided by comparison with Hematoxylin and Eosin stained near or adjacent sections (Figures 1A and 1B). This simple classification scheme allowed for an unbiased analysis of all Y-chromosome-containing cells within the epithelial and non-epithelial compartments. Retrospective staining for cytokeratin, a recognized epithelial-specific marker, confirmed the selectivity of our unbiased compartmentalization strategy (Figure 1B, inset). We quantified the number of X-chromosomes present within Y-chromosome-positive, Lamin B1-circumscribed nuclei in both epithelial and non-epithelial compartments in intestinal epithelial biopsies from gender-mismatched transplant patients and normal male controls (Figure 1 and Table 2).

**Table 1:** Patient characteristics and detection of Y-chromosome positive epithelial cells.

								number of epithelial cells		
									with Y-chromosome	
Patient	Gender	Donor gender	Reason for transplant*	Type of trans-plant**	Days from transplant to biopsy	Biopsy Site	Pathological diagnosis***	analyzed	Total	With ≥2 X chromosomes
1	F	M	ALL	PBSC	86	duodenum	GVHD	4200	10	3
2	F	M	NHL	PBSC	49	colon	GVHD	2100	1	1
3	F	M	AML	PBSC	30	duodenum	GVHD	3300	17	3
4	F	M	MDS	PBSC	25	small bowel	GVHD	3900	25	1
5	F	M	CML	BM	60	sigmoid colon	GVHD	2700	10	1
6	F	M	AML	PBSC	28	sigmoid colon	GVHD	3900	2	1

* ALL, acute lymphoblastic leukemia; NHL, non-Hodgkin lymphoma; AML/CML, acute/chronic myeloid leukemia; MDS, myelodysplastic syndrome.

** PBSC, peripheral blood stem cells; BM, bone-marrow.

*** GVHD, graft versus host disease.

**Table 2:** Sex karyotypes of epithelial and non-epithelial cells.

	# of cells with observed sex karyotype			
	Y	XY	XXY	XXXY
normal male epithelium	25	47	2	0
normal male non-epithelium	25	53	1	0
transplant epithelium	15	40	9	1
transplant non-epithelium	31	36	1	0

### Validation of staining and scoring strategy

In normal male intestinal sections the majority of epithelial and non-epithelial nuclei harbored a single Y-chromosome and a single X-chromosome, as expected (Table 2). Approximately one-third of Y-chromosome-containing nuclei did not contain an X-chromosome, likely an artifact of analysis within 5 µm tissue sections, which include partial nuclei. Rare instances of nuclei with one Y-chromosome and two X-chromosomes were also detected (Table 2). In these normal male intestinal sections, we observed that epithelial and non-epithelial nuclei had similar distributions of sex karyotypes, consistent with the expectation that the overwhelming majority of cells in each compartment were diploid (Table 2). To evaluate FISH probe specificity, we also stained intestinal tissue sections from two female patients with GVHD that had received gender-matched hematopoietic cell transplant, and observed no nuclear Y-chromosome signals in more than 7,000 cells (not shown). These experiments demonstrate the efficiencies of X- and Y-chromosome FISH staining and establish a baseline X-chromosome number distribution in normal male intestinal epithelial and non-epithelial cells.

### Increased X-chromosome number in donor-marker-carrying intestinal epithelial nuclei

**Figure 2:**
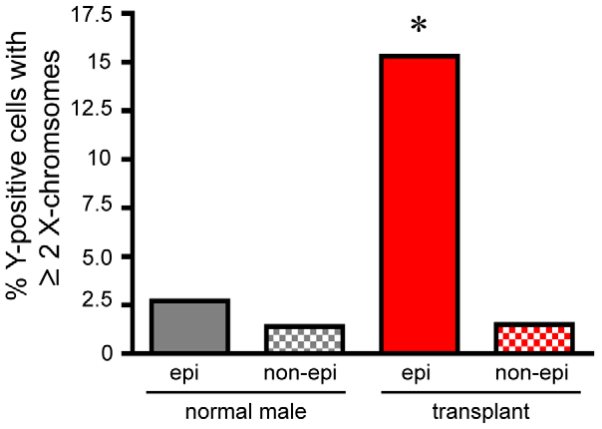
Frequency of nuclei with 2 or 3 X-chromosomes. Percent of Y-chromosome-positive nuclei with two or three X-chromosomes, observed in epithelial (epi) and non-epithelial (non-epi) compartments of normal male and male-into-female gender mismatched bone marrow transplant patients (transplant). *, p = 0.0016, Fisher's exact test.

To examine the occurrence of cell-fusion between hematopoietic and epithelial cells in human intestine, we quantified the number of X-chromosomes in Y-chromosome-positive nuclei of intestinal tissue from female patients who had received gender-mismatched hematopoietic cell transplant. We scored only nuclei in which X- and Y-chromosomes were clearly contained within a well demarcated contiguous nuclear envelope, as marked by Lamin B1 staining (Figure 1B–1D). In the non-epithelial compartment, Y-chromosome-positive nuclei represent donor-derived blood cells within the intestinal mesenchyme, their expected histological location. While the frequency of donor-derived cells in the mesenchyme varied between transplant patients (not shown), only a small fraction of these nuclei had more than a single X-chromosome (1.5%), a similar proportion as observed in normal male epithelial (2.7%) or non-epithelial populations (1.3%) (Table 2 and Figure 2). This result is consistent with the identity of these cells as normal diploid donor-derived blood cells. By contrast, analysis of more than 20,000 nuclei in the epithelial compartment of six female patients that had received male bone marrow identified sixty-five nuclei carrying Y-chromosomes (Table 1) and ten of these (15%) also had two or more X-chromosomes (Table 2 and Figure 2). Nine of these nuclei had two X-chromosomes and one nucleus had three X-chromosomes (examples in Figure 1C and 1D); nuclei with two or more X-chromosomes were identified in all patient samples (Table 2). Statistical comparison of the incidence of nuclei with 2 or more X-chromosomes between cell populations yielded a p-value of 0.0016, indicating a much higher incidence of nuclei with 2 or more X-chromosomes in Y-chromosome positive epithelial cells of transplant patients than in any other cell population (Figure 2). These results support the hypothesis that donor-marker-carrying cells within the epithelium of hematopoietic cell transplant recipients comprise a karyotypically distinct cell population, and are highly consistent with their origin as a result of fusion between hematopoietic lineage cells and intestinal epithelial cells.

### Differentiation status of epithelial regions carrying fusion-derived cells

**Figure 3:**
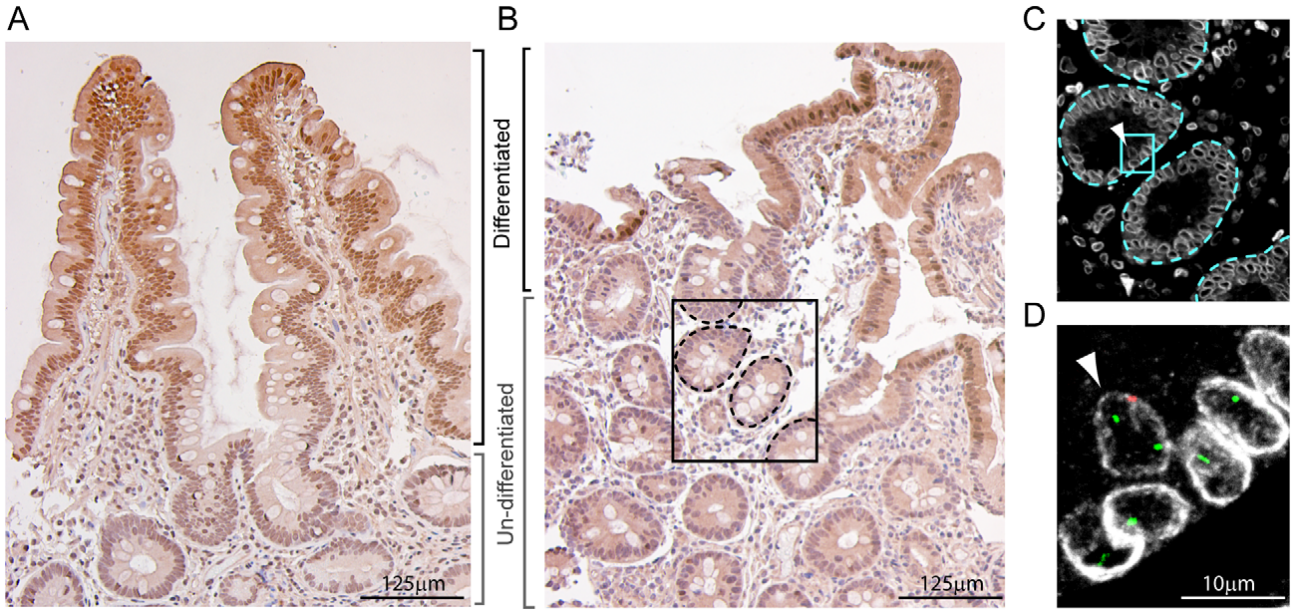
Differentiation status of cells with abnormal sex-karyotypes. **(**A) FABP2/IFABP expression in a control and (B) transplant patient sample with an example of an XXY cell. Brackets indicate differentiated (high Fabp2/Ifabp expression, black brackets) and undifferentiated (low Fabp2/Ifabp expression, gray brackets) regions of epithelium within each sample. (C) Enlarged view of boxed region from *B*, in an adjacent tissue section stained for Lamin B1.(D) Enlarged view of boxed region from panel C, showing X- (green) and Y- (red) chromosomes and Lamin B1 (white). Arrowhead indicates the same nucleus in panels C and D. Dashed lines indicate boundaries of epithelial and non-epithelial compartments.

Studies in mice suggest that hematopoietic lineage cells fuse directly with stem or long-lived proliferating progenitors in the intestine [14]. To determine whether this was also the case in humans, we analyzed adjacent tissue sections to the FISH-stained sections for the expression of the intestinal fatty acid binding protein (Fabp2/Ifabp), a marker of differentiated intestinal epithelial cells [20] (Figure 3A). Ifabp expression was low or absent in epithelial regions containing cells with abnormal sex-karyotypes—cells likely derived from fusion—indicating that hematopoietic-lineage cells had fused with undifferentiated and proliferating epithelial progenitors (Figure 3).

## Discussion

Previous work has demonstrated that genetic markers specific to transplanted hematopoietic cells can often be found in non-hematopoietic tissue of transplant recipients. Using an unbiased and direct quantitative and statistical approach to evaluate the Y-chromosome-containing population of cells in female gender-mismatched hematopoietic cell transplant patients, we now provide evidence that cell fusion is one mechanism by which this occurs. In these patients, a genetic marker which should be exclusively found within hematopoietic lineage cells—the Y-chromosome—is detectable in cells of the epithelium. If this resulted exclusively from hematopoietic transdifferentiation or from intra-epithelial invasion of lymphocytes, as previously suggested, these cells should have a similar number of X-chromosomes as normal diploid male cells [18]. However, within this population we find a statistically significant increase in the number of cells with 2 or more X-chromosomes. This indicates that these cells are derived from hematopoietic-epithelial cell fusion, since only fusion—and neither transdifferentiation nor intra-epithelial lymphocytic invasion—directly produces cells with an increased chromosome number. Many of the Y-chromosome-containing epithelial nuclei we identified in hematopoietic cell transplant patients had either zero or one X-chromosome. These cells could either be intra-epithelial donor lymphocytes, examples of hematopoietic transdifferentiation, fusion-derived cells that lost X-chromosomes during mitosis, or partial nuclei of fused cells that were incompletely analyzed due to tissue sectioning limitations.

It is important to note that our analysis was focused on determining whether hematopoietic-epithelial fusion occurs in human intestinal tissue, independent of the phenotypic outcomes of fusion. Similar to an analysis of fusion between hematopoietic cells and Purkinje neurons, we used a robust molecular marker independent method to classify cellular compartments [4]. Previously published studies have analyzed intestinal tissue for evidence of cell fusion by relying on cell type specific marker expression. However, restricting the identification of fusion events to cells that express specific markers—either hematopoietic or epithelial—may inadvertently exclude fusion-derived cells, biasing analysis. Of course, retrospective analysis of protein levels or gene expression in fusion-derived cells identified by more direct methods is important for understanding phenotypes of these cells and their impact on tissue physiology. Notably, we observed that at least a subset of XXY cells expressed the epithelial marker cytokeratin, suggesting that fusion-derived cells are likely phenotypically *similar* to adjacent epithelial cells (Figure 1B, inset). It remains unclear whether the combination of hematopoietic and epithelial genomes by cell fusion results in cells that are phenotypically *identical* to other epithelial cells. In mice, cells derived from fusion between hematopoietic and intestinal epithelial cells have altered gene expression patterns relative to normal epithelium [16]. Similarly, hepatocytes derived from hematopoietic-hepatocyte cell fusion also are transcriptionally distinct from normal hepatocytes [21]. In the intestine, these transcriptional differences could result in altered levels of traditional epithelial proteins, complicating cell identification based on molecular markers. Although we have previously performed sequence based transcriptional analysis of the cellular products formed by *in vivo* hematopoietic-epithelial fusion in mice, there is insufficient data at the single-cell level to predict the fates of these cells and the degree to which they have altered expression of lymphocyte or epithelial cell surface markers [16].

While the presence of donor cells with supernumerary X-chromosomes in the epithelium of hematopoietic cell transplant patients almost certainly reflects cell fusion, there are several less probable explanations. Our data could reflect the preferential epithelial invasion or transdifferentiation of hematopoietic cells with an increased chromosome number. Alternatively, it is also possible that lymphocytes which invade into the epithelium are prone to high rates of chromosome segregation errors, resulting in the gain of additional X-chromosomes. However, there is neither experimental support nor mechanistic basis for either of these possibilities. By contrast, there is strong evidence for cell fusion between hematopoietic and non-hematopoietic cells in multiple tissue types in mice and also for a degree of hematopoietic cell fusion with neurons in humans [4], [6], [12]-[14], [22]. Therefore, our data and analyses demonstrate the occurrence of hematopoietic-epithelial fusion in the human intestine, providing the first definitive evidence for hematopoietic cell fusion with any non-neoplastic epithelial cell type in humans.

In mice, hematopoietic cells can fuse with proliferating intestinal stem cells, and our data suggest that the same occurs in humans [14], [17]. Specifically, the detection of individual cells with XXY and XXXY sex-karyotypes is highly consistent with these cells arising from the proliferation of fused cells. If hematopoietic fusion occurred with terminally differentiated epithelial cells, then this would produce binucleated cells. Each nucleus would remain diploid since these cells are post-mitotic and the only recognized path from binucleation to mononucleation is mitosis. The identification of a significant increase in the incidence of cells with 2 or more X-chromosomes *per* in Y-chromosome-positive epithelial cells in gender mismatched transplant patients therefore indicates that fusion occurs between hematopoietic lineage and progenitor cells within the proliferative zone of the intestine. Consistent with this, Y-chromosome positive epithelial cells with supernumerary X-chromosomes were detected within the proliferative crypt compartment as determined by lack of Ifabp expression, a marker of epithelial differentiation.

Establishing hematopoietic cell fusion with non-hematopoietic cell types in humans provides the basis for subsequent analysis of the physiological function and pathological potential of fusion between different cell types. In mice, hematopoietic fusion with non-hematopoietic cell types occurs in the absence of overt inflammation or tissue injury associated with hematopoietic transplant, and hematopoietic fusion with non-hematopoietic cell types in humans therefore likely occurs endogenously in the absence of disease [6], [17], [23]. Currently, the phenotypes of fusion-derived cells are poorly understood and therefore the physiological consequences of fusion between different cell types remain unclear. In the brain, fusion-derived neurons are rare and non-proliferative and so there is limited potential for the expansion of a population of these cells [4], [7]. It remains untested whether hematopoietic cell fusion with Purkinje neurons alters normal Purkinje function, and observations of an increased numbers of fusion-derived Purkinje neurons in ageing mice and in pathologic mouse and human brains provide the impetus for additional study [7], [24], [25]. In the intestine, there is high potential for a physiological impact of cell fusion. In mice, hematopoietic lineage cells fuse with intestinal epithelial progenitors, resulting in the long-term production of progeny of a single fusion event [14]. We find that the same occurs in humans. Further, the gene expression profile of fusion-derived cells shows ongoing transcription of hematopoietic genes, suggesting these cells may be phenotypically unique [16]. It is now critically important to understand the phenotypic consequences of spontaneous fusion between hematopoietic lineage and epithelial cells in order to understand the physiological relevance of hematopoietic-epithelial cell fusion in the intestine and other human tissues.
